# Structural insights into a high fidelity variant of SpCas9

**DOI:** 10.1038/s41422-018-0131-6

**Published:** 2019-01-21

**Authors:** Minghui Guo, Kuan Ren, Yuwei Zhu, Ziyun Tang, Yuhang Wang, Bailing Zhang, Zhiwei Huang

**Affiliations:** HIT Center for Life Sciences, School of Life Science and Technology, Harbin Institute of Technology, Harbin, Heilongjiang 150080 China

**Keywords:** Molecular biology, X-ray crystallography

## Abstract

The RNA-guided endonucleases of the CRISPR-Cas9 system, including the most widely used Cas9 from *Streptococcus pyogenes* (SpCas9), are becoming a robust genome editing tool in model organisms and hold immense promise for therapeutic applications. Many strategies have been employed to overcome the limitations caused by SpCas9’s off-target effects and its stringent requirement for the protospacer adjacent motif (PAM) sequence. However, the structural mechanisms underlying these strategies remain undefined. Here, we present crystal structure of a SpCas9 variant, xCas9 3.7 that has broad PAM compatibility and high DNA targeting specificity, in complex with a single-guide RNA and its double-stranded DNA targets. Structural comparison revealed that salt bridge-stabilized R1335 is critical for the stringent selection of PAM sequence by SpCas9. Unrestricted rotamerization of this residue by the E1219V mutation in xCas9 3.7 lessens the stringency for PAM recognition and allows SpCas9 to recognize multiple PAM sequences as further supported by biochemical data. Compared to those in wild-type (WT) SpCas9, REC2 and REC3 domains in xCas9 3.7 undergo striking conformational changes, leading to reduced contact with DNA substrate. SpCas9 mutants engineered to display less interaction with DNA and have conformationally more flexible REC2 and REC3 domains display enhanced specificity for DNA substrates in both biochemical and cellular assays. Taken together, our findings reveal the structural mechanisms underlying the broadened PAM compatibility and high DNA fidelity of xCas9 3.7, which can assist rational engineering of more efficient SpCas9 variants and probably other Cas9 orthologs.

## Introduction

Most of archaea and many bacteria encode CRISPR-Cas (clustered regularly interspaced short palindromic repeats and CRISPR-associated proteins) adaptive immune systems to defend themselves from phage invasion.^1–5^ In the type II CRISPR-Cas9 system, the crRNA (CRISPR RNA):tracrRNA (trans-activating crRNA)-guided Cas9 degrades double-stranded DNA (dsDNA) bearing a PAM (protospacer-adjacent motif) and an adjacent 20-nt sequence complementary to the crRNA.^6–11^ The Cas9 protein comprises a recognition (REC) lobe and a nuclease (NUC) lobe.^8–10^ The REC lobe is composed of three REC domains (REC1-3) to recognize the guide RNA scaffold and guide RNA/DNA heteroduplex. Two endonuclease domains (HNH and RuvC) in the NUC lobe connected by an arginine-rich bridge helix (BH) and a PAM-interacting (PI) domain are used to cleave the target and non-target strand of dsDNA, respectively. The sgRNA (single-guide RNA)-guided *S. pyogenes* Cas9 (SpCas9) system has been harnessed as the most widely used tool for genome manipulation, such as target gene disruption, transcriptional repression and activation, epigenetic modulation, and single base-pair conversion in various organisms and cell types.^12–17^

PAM compatibility and off-target effects are two major limitations that hinder potential therapeutic applications of the SpCas9 system. Several strategies for engineering SpCas9 to overcome the limitations have been reported.^18–21^ For example, the high fidelity SpCas9 variants SpCas9-HF1, eSpCas9 and HypaCas9 were made through multiple mutations of DNA-interacting residues of SpCas9 to reduce the energetics of target DNA recognition and cleavage.^19–21^ More recently, the phage-assisted continuous evolution (PACE) method^22^ was used to identify a couple of SpCas9 variants recognizing multiple PAM sequences. One of the variants is called xCas9 3.7 (carrying 7 point mutations, A262T, R324L, S409I, E480K, E543D, M694I and E1219V, compared with wild-type (WT) SpCas9) with the broadest compatibility for 5′-NG-3′, 5′-GAA-3,′ and 5′-GAT-3′ PAM sequences in mammalian cells.^22^ Remarkably, in addition to expanded PAM compatibility, xCas9 3.7 has much greater substrate specificity and substantially lower off-target effect at both 5′-NGG-3′ and non-5′-NGG-3′ PAM sites in human cells. Thus, the xCas9 3.7 variant represents a collective of high editing efficiency, broad PAM compatibility and high DNA targeting specificity. However, the molecular mechanisms of the broadened PAM recognition and improved DNA specificity of xCas9 3.7 remain unknown.

## Results

### The overall structural comparison of xCas9 3.7 with WT SpCas9

To provide structural insights into the molecular mechanisms underlying expanded PAM recognition and improved cleavage fidelity of xCas9 3.7, we determined the crystal structures of xCas9 3.7 in complex with a 100-nucleotides (nt) sgRNA, a 28-nt target DNA strand and an 11-nt non-target DNA strand containing either 5′-GAT-3′ PAM or 5′-AAG-3′ PAM sequence, at 2.7 and 3.0 Å resolutions, respectively (Supplementary information, Fig. S1a, b and Supplementary information, Table S1). Since the two structures are virtually identical (root-mean-square deviation [RMSD] of 0.29 Å for 1208 equivalent Cα atoms) (Supplementary information, Fig. S1c), we mainly discuss the quaternary complexstructure containing the 5′-GAT-3′ PAM unless otherwise stated.

**Fig. 1:**
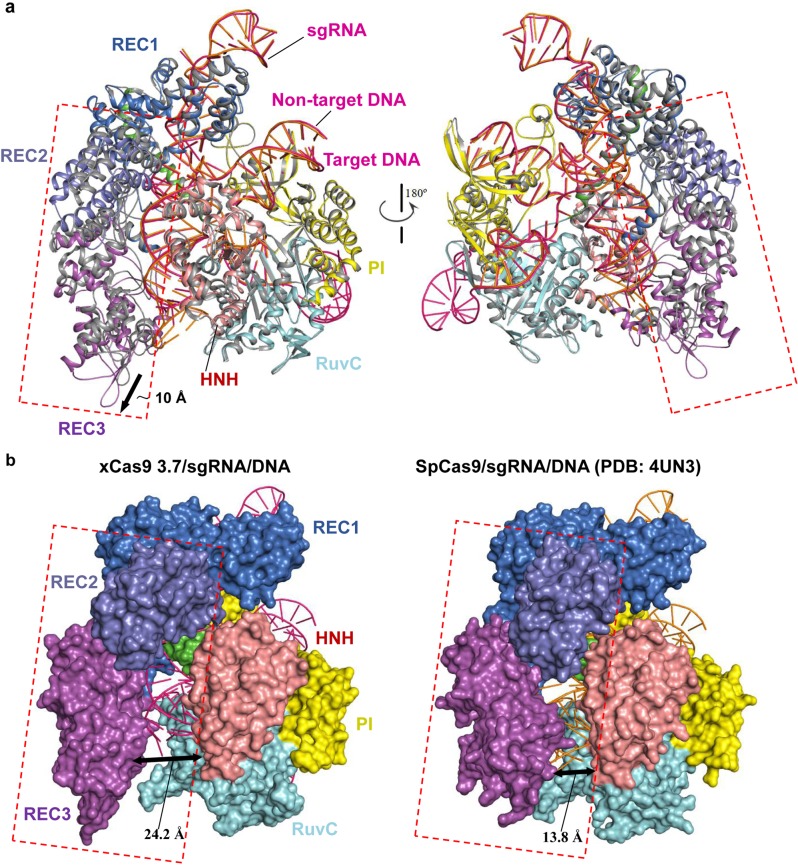
Structural comparison of xCas9 3.7 with WT SpCas9. **a** Superimposition of the overall structures of xCas9 3.7/sgRNA/DNA and WT SpCas9/sgRNA/DNA (PDB: 4UN3). The REC1, REC2, REC3, RuvC, BH, HNH, and PI domains of xCas9 3.7 are colored in blue, lightblue, purple, cyan, green, salmon, and yellow, respectively. The xCas9 3.7- and WT SpCas9 (gray)-bound sgRNA/DNA are colored hot pink and orange, respectively. **b** Overall structural comparison of xCas9 3.7/sgRNA/DNA and WT SpCas9/sgRNA/DNA (PDB: 4UN3). Domains with conformational changes are indicated by dashed lines

Structural comparison between xCas9 3.7 and SpCas9 revealed that, despite their overall similar architectures, there are significant conformational differences in REC2 and REC3 domains relative to the NUC lobe (Fig. 1a). Specifically, compared to that in SpCas9, the REC3 domain of xCas9 3.7 at the proximal end of the REC lobe moves about 10 Å away from the guide RNA/DNA heteroduplex, leaving the 5′ end of the RNA/DNA heteroduplex solvent exposed (Fig. 1b). The REC2 domain of xCas9 3.7 also undergoes substantial structural rearrangement. It rotates away from the guide RNA/DNA heteroduplex and the REC1 domain to stabilize the conformation of REC3 domain (Fig. 1b). Despite the conformational changes in REC2 and REC3 domains, they remain in contact with each other at the PAM-distal end, implying that these conformational changes in xCas9 3.7 may play an important role in enhancing DNA targeting specificity, which is discussed in detail below.

### Recognition of PAM sequences by xCas9 3.7

**Fig. 2:**
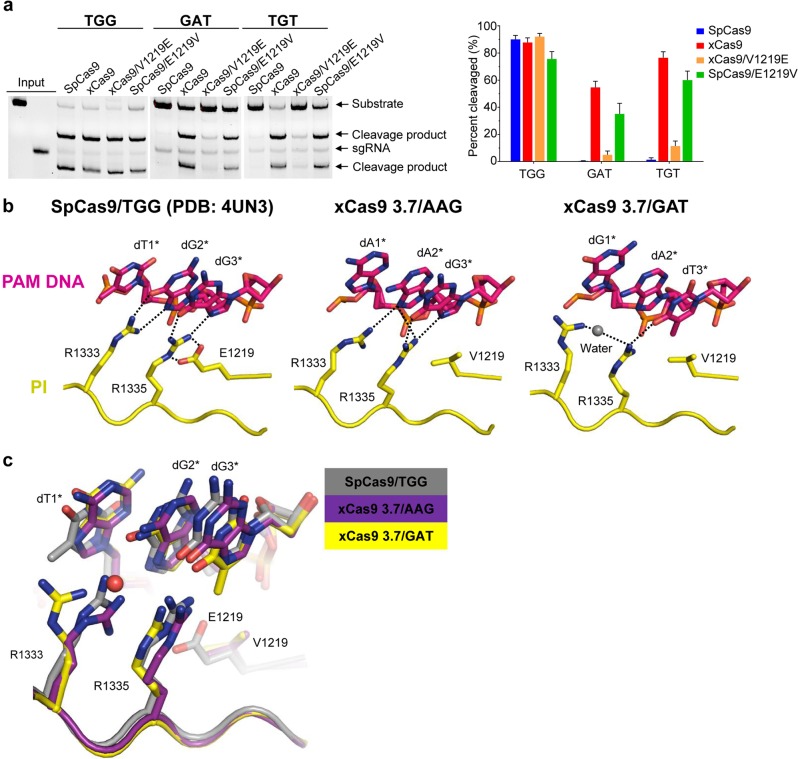
5′-GAT-3′ and 5′-AAG-3′ PAM recognition by xCas9 3.7. **a** Left: In vitro cleavage assays of SpCas9, xCas9 3.7 and xCas9 3.7 variants using substrates containing different PAM sequences. The gels were resolved by EB staining following denaturing TBE-Urea PAGE. Data shown is representative of three independent experiments. Right: Quantification of in vitro cleavage activity (shown in left). **b** Zoom-in views of 5′-TGG-3′ PAM recognition by SpCas9 (left panel) and 5′-AAG-3′ and 5′-GAT-3′ PAM recognition by xCas9 3.7 (middle and right panels). **c** Close-up view of 5′-GAT-3′ and 5′-AAG-3′ PAM-bound xCas9 3.7 superimposed with 5′-TGG-3′ PAM-bound SpCas9. Color codes are indicated

Among the seven amino acids substituted in the xCas9 3.7 variant, V1219 is the only residue located in the PI domain (Supplementary information, Fig. S1b), suggesting that this residue is a structural determinant for PAM recognition in xCas9 3.7. In support of this idea, an SpCas9 mutant carrying the single residue mutation E1219V displayed a significantly enhanced activity in cleaving substrates containing 5′-GAT-3′ or 5′-TGT-3′ PAM as compared to WT SpCas9 (Fig. 2a). Conversely, xCas9 3.7 with V1219 changed back to Glu greatly reduced its catalytic activity toward the same two substrates. These biochemical data support an important role of E1219 in determining the PAM specificity of SpCas9. Interestingly, however, structural superposition of 5′-GAT-3′ PAM-bound xCas9 3.7 (referred to as xCas9 3.7/GAT) or 5′-AAG-3′ PAM-bound xCas9 3.7 (referred to as xCas9 3.7/AAG) with WT SpCas9 revealed that their PI domains have nearly identical conformations (Supplementary information, Fig. S1d). This structural observation indicates that conformational changes in the PI domain of xCas9 3.7 are unlikely to be responsible for their broader PAM recognition.

In the structure of WT SpCas9^8^ (PDB ID 4UN3), E1219 establishes a pair of salt bridges with R1335 and maintains its stability (Fig. 2b). The E1219-stabilized R1335 and its neighboring R1333 form Watson-Crick-like hydrogen bonds with dG3* and dG2* of 5′-TGG-3′ PAM, respectively, thus defining the base-specific recognition of GG by WT SpCas9. Mutation of E1219 to Val disrupts salt bridges with R1335, making it possible for the latter residue to adopt multiple rotamer conformations. Indeed, the side chain of R1335 in the structure of xCas9 3.7/GAT adopts a different rotamer conformation from that in WT SpCas9, making a hydrogen bond with the nucleobase of dT3* (Fig. 2b, c and Supplementary information, Fig. S2). Because of salt bridges with E1219, changes in rotamer of R1335 in WT SpCas9 are greatly restrained. Structural comparison revealed that the methyl group of dT1* from the substrate dGAT clashes with R1335 in WT SpCas9, thus making dGAT a poor substrate of the WT SpCas9 system (Fig. 2c). The relaxed R1335 in xCas9 3.7 also allows the side chain of R1333, which packs tightly against the E1219-stabilized R1335 in WT SpCas9, to move more freely. Consistently, compared to that in WT SpCas9 structure, the side chain of R1333 in the structure of xCas9 3.7/AAG rotates about 30 degrees to contact the nucleobase of dA2* (Fig. 2b, c). Taken together, our results show that the rotamer flexibility of R1335 is an important determinant for the recognition specificity of PAM by SpCas9.

### Conformational rearrangement in REC2 and REC3 domains of xCas9 3.7

**Fig. 3:**
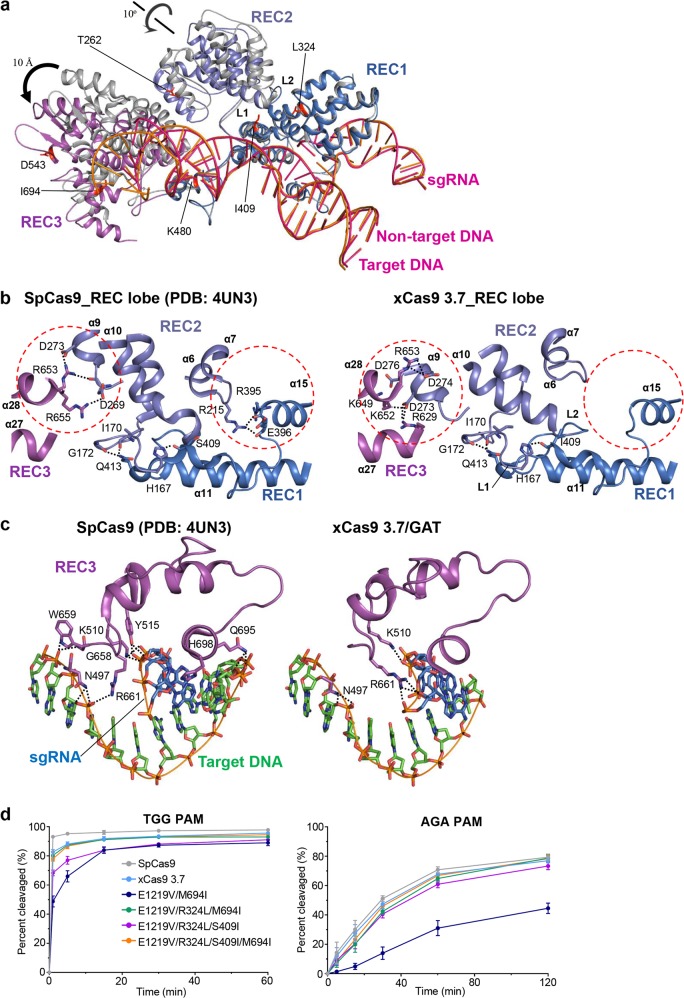
Structural rearrangement of the REC lobe of xCas9 3.7. **a** Superimposition of the RNA/DNA heteroduplex-bound REC lobe of SpCas9 (PDB: 4UN3) and xCas9 3.7. Color codes are the same as those shown in Fig. 1a. The six residues in REC lobe substituted in xCas9 3.7 are highlighted in red. **b** A close-up view of the detailed interactions of REC domains of SpCas9 (shown in left) and xCas9 3.7 (shown in right). Black dashed lines represent hydrogen bonds. **c** A close-up view of the detailed interactions between REC3 and RNA/DNA heteroduplex of SpCas9 (shown in left) and xCas9 3.7 (shown in right). **d** Quantification of in vitro cleavage activity of xCas9 3.7 variants using substrates containing different PAMs. *n* = 3. Error bars represent S.D

Aside from the contacts between PAM and SpCas9, a large majority of SpCas9-DNA interaction is mediated by the REC2 and REC3 domains (Fig. 3a and Supplementary information, Fig. S3). Structural comparison revealed that these two domains in xCas9 3.7, relative to those in SpCas9, rotate about 10 Å away from the RNA/DNA heteroduplex (Fig. 3a, b). To exclude the possibility of the crystal packing effect-induced structural rearrangement, we compared the crystallographic parameter of SpCas9-sgRNA-DNA (PDB: 4UN3) with that of xCas9 3.7 and found that the two structures have the same space group and similar lattice parameters. As shown in the supplementary figure S4, the two structures adopt the same crystal packing arrangement, further supporting that the observed structural rearrangement in REC2 and REC3 domains of xCas9 3.7 is induced by the mutations in xCas9 3.7. This striking conformational change substantially reduces the buried surface of the bound DNA, from 8075.2 Å^2^ in SpCas9 to 6649.2 Å^2^ in xCas9 3.7 (Fig. 3c). Due to the structural rearrangement in REC3 domain, residues Y515, G658, W659, Q695 and H698 supposed to interact with the RNA/DNA heteroduplex in SpCas9^8^ make no interaction with that in xCas9 3.7 (Fig. 3c). Despite the striking conformational change in REC2 and REC3 domains, the RNA/DNA heteroduplex in the two structures adopts a highly conserved conformation except that the three nucleobases from the 3′ end of the target DNA strand in xCas9 3.7 are invisible due to flexibility (Supplementary information, Figs. S3, S5a and b). Notably, in the structure of RNA/DNA-bound SpCas9, M694 forms van der Waals contacts with the last two nucleobases of the DNA substrate, and the interaction would be compromised by the M694I mutation in xCas9 3.7 (Supplementary information, Fig. S6). R324 and S409, located at the REC1-REC2 interface, are involved in intra-molecular interactions, and mutations of these two residues (R324L and S409I) can perturb the conformation of REC3 domain that is employed by SpCas9 to interact with the 3′ end of the DNA substrate (Supplementary information, Fig. S6). Thus, all the three mutations M694I, R324L and S409I appear to act to compromise interactions between SpCas9 and the DNA substrate. Together with the previously reported data showing that the specificity of SpCas9 can be enhanced through mutations specifically impairing SpCas9-DNA interactions,^20–22^ our structural observation guides us to investigate the effect of the conformational changes in REC2 and REC3 domains contributing to the reduced off-target cleavage of xCas9 3.7.

We next sought to determine which residues among the 7 residues substituted in xCas9 3.7 are responsible for the observed enhancement of DNA targeting specificity without sacrificing on-target cleavage efficiency. The observation that the E1219V mutation is required for the broader PAM compatibility of xCas9 3.7 suggests that the remaining six mutations (A262T, R324L, S409I, E480K, E543D and M694I) should contribute to the high DNA targeting specificity of xCas9 3.7. Interestingly, all the 6 mutations are located in the REC lobe, which display striking conformational differences from that of WT SpCas9. Further structural inspection showed that A262T, E480K and E543D are solvent-exposed and not involved in intra-molecular interactions or contacts with the RNA/DNA heteroduplex (Fig. 3a), suggesting that these three mutations may not be important for the DNA targeting specificity of xCas9 3.7. Supporting this hypothesis, a SpCas9 mutant with the mutations E1219V, R324L, S409I and M694I exhibited a comparable efficiency with xCas9 3.7 in cleaving the 5′-TGG-3′ and 5′-AGA-3′ PAM substrates (Fig. 3d). However, the E1219V/M694I mutant had a severe effect on the efficiency of SpCas9 in processing substrates with 5′-TGG-3′ or 5′-AGA-3′ PAM, and the E1219V/R324L/S409I mutant displayed a reduced efficiency in cleaving 5′-TGG-3′ PAM-containing substrates (Fig. 3d). These data indicate that mutations in the two high fidelity hotspot interfaces of REC1-REC2 (HFH1, including residues of R324 and S409) and REC3-RNA/DNA heteroduplex (HFH2, including residue M694) (Supplementary information, Fig. S6) coordinately improved cleavage fidelity.

### Structure-based high-fidelity SpCas9 variant design

**Fig. 4:**
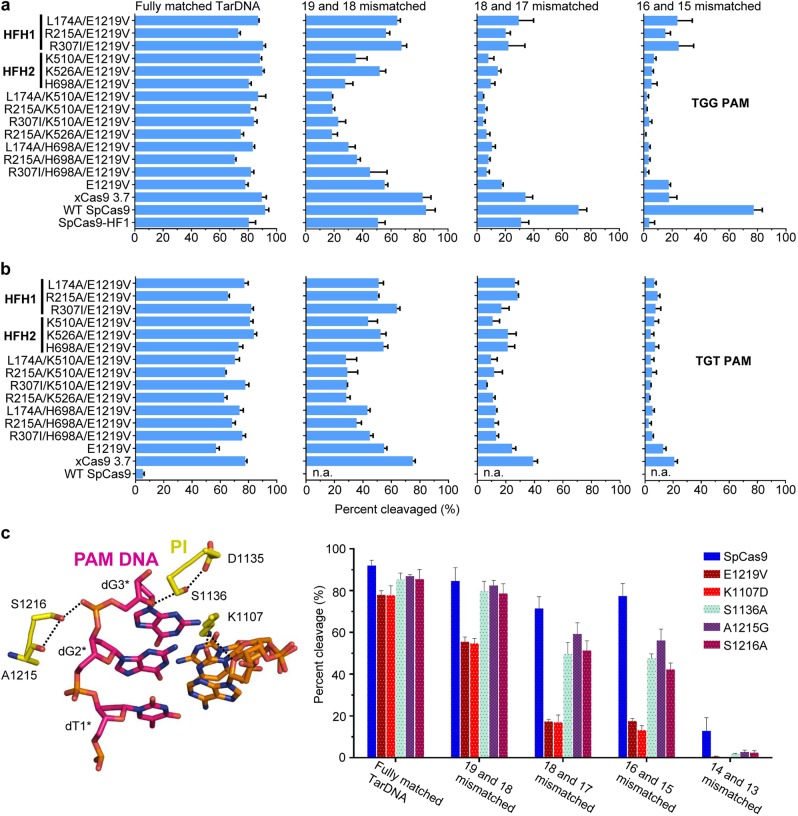
Structure-based design of high-fidelity SpCas9 variants. **a** In vitro cleavage assay showing the substrate specificity of SpCas9 variants. Activities of rationally designed SpCas9 variants using fully matched or doubly mismatched DNA substrates with 5′-TGG-3′ PAM. *n* = 3. Error bars represent S.D. **b** In vitro cleavage assay showing the substrate specificity of SpCas9 variants. Activities of rationally designed SpCas9 variants using fully matched or doubly mismatched DNA substrates with 5′-TGT-3′ PAM. *n* = 3. Error bars represent S.D. **c** Mutations at PAM recognition sites increase SpCas9 substrate specificity. Left: Zoom-in view of the 5′-TGG-3′ PAM DNA recognition by SpCas9. Right: Activities of structure-based mutagenesis of SpCas9 (see left) for fully matched or doubly mismatched DNA substrates containing 5′-TGG-3′ PAM. *n* = 3. Error bars represent S.D

Our structural data showed that mutations in HFH1 and HFH2 region of xCas9 3.7 lead to a diminished DNA binding surface as compared to WT SpCas9, raising the possibility that disturbing the conformational stabilization of REC lobe thus weakening the DNA-binding ability of SpCas9 could be applied to rationally design novel high-fidelity SpCas9 variants. To further test this hypothesis, we engineered substitutions in SpCas9 and investigated the activity of resulting mutants to cleave different DNA substrates. The substitutions were made in the E1219V mutant because of its important role in broadening PAM recognition of SpCas9. We first examined the activity of mutants carrying substitutions of K510, K526 or H698 located in HFH2 interface that are directly involved in the interaction with DNA (Supplementary information, Fig. S6). Single mutations in one of the three residues (K510A, K526A or H698A) coupled with E1219V had little effect on the catalytic activity of SpCas9 in processing substrates with 5′-TGG-3′ or 5′-TGT-3′ PAM (Fig. 4a, b). However, all of the three mutants (K510A/E1219V, K526A/E1219V and H698A/E1219V) displayed lower catalytic activity toward mismatched substrates compared with WT SpCas9 (Fig. 4a, b), indicating that mutations of these residues enhanced the stringency of substrate recognition of SpCas9. We then tested the effect of mutations of residues involved in stabilization of the conformation of REC2 and REC3 domains on the activity of E1219V mutant. We reasoned that L174A, R215A and R307I mutations located at the HFH1 interface would not affect the structural integrity of REC2 and REC3 domains but promote their conformational flexibility (Supplementary information, Fig. S6). Indeed, these mutants suppressed off-target effects of xCas9 3.7 while keeping its on-target cleavage activity comparable to SpCas9 (Fig. 4a, b). Remarkably, combined mutations of L174A/K510A/E1219V, R215A/K510A/E1219V, R307I/K510A/E1219V, R215A/K526A/E1219V, L174A/H698A/E1219V, R215A/H698A/E1219V and R307I/H698A/E1219V at REC1-REC2 and REC3-RNA/DNA heteroduplex interfaces further lowered the off-target cleavage activity toward substrates containing 5′-TGG-3′ or 5′-TGT-3′ PAM (Fig. 4a, b). Surprisingly, the single mutation of E1219V also displayed decreased off-target cleavage activity (Fig. 4a, b), suggesting that perturbing PI domain-mediated PAM sequence recognition can also enhance the DNA targeting specificity of SpCas9. We therefore made SpCas9 variants K1107D, S1136A, A1215G or S1216A predicted to be compromised in their PAM recognition. As shown in Fig. 4c, one of these variants, SpCas9 K1107D displayed an activity profile similar to SpCas9 E1219V in cleaving matched and mismatched substrates, indicating that this SpCas9 mutant also has enhanced substrate specificity. This result is consistent with a previous study in human cells.^23^

### Rationally designed SpCas9 variants display enhanced DNA specificity in human cells

**Fig. 5:**
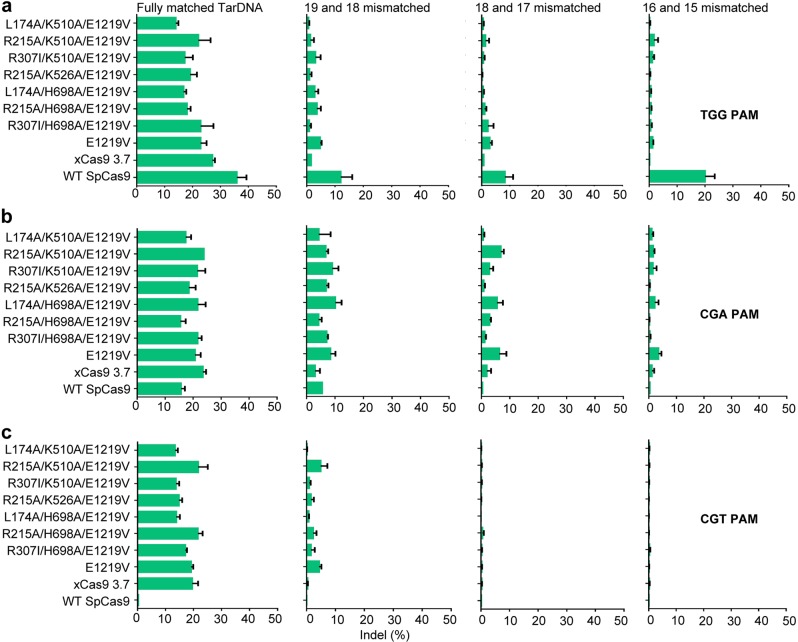
Rationally designed SpCas9 variants have higher specificity in human cells. **a** Analysis of the DNA targeting specificity of rationally designed SpCas9 variants in human cells. Frequencies of Indels induced by rationally designed SpCas9 variants guided by doubly mismatched sgRNAs against *EMX1* site bearing 5′-TGG-3′ PAM quantified by T7E1 assay. *n* = 3. Error bars represent S.D. **b** Analysis of the DNA targeting specificity of rationally designed SpCas9 variants in human cells. Frequencies of Indels induced by rationally designed SpCas9 variants guided by doubly mismatched sgRNAs against *EMX1* site bearing 5′-CGA-3′ PAM quantified by T7E1 assay. *n* = 3. Error bars represent S.D. **c** Analysis of the DNA targeting specificity of rationally designed SpCas9 variants in human cells. Frequencies of Indels induced by rationally designed SpCas9 variants guided by doubly mismatched sgRNAs against *EMX1* site bearing 5′-CGT-3′ PAM quantified by T7E1 assay. *n* = 3. Error bars represent S.D

We next tested the above rationally designed SpCas9 variants in human cells for their cleavage accuracy. To this end, we mutated the guide RNA targeting the *EMX1* gene (Supplementary information, Table S2) to introduce double-base mismatches at different positions (Fig. 5a). Consistent with the biochemical data (Fig. 4a), SpCas9 variants with combined mutations at HFH1 and HFH2 interfaces exhibited much lower levels of indel with mismatched guides than WT SpCas9 at 5′-TGG-3′ PAM site (Fig. 5a). Furthermore, these mutants displayed greater or similar DNA targeting specificity toward substrates with 5′-CGA-3′ and 5′-CGT-3′ PAM sites than xCas9 3.7, while possessing comparable on-target activity compared with xCas9 3.7 (Fig. 5b, c).

## Discussion

Although the stringent requirement for the PAM sequence enables the CRISPR-Cas immune system to efficiently distinguish ‘self’ from ‘non-self’, it restricts the selection of targetable sequences in the applications of genome editing. SpCas9 has been proved to be a powerful tool for genome editing, which specifically recognizes the 5′-NGG-3′ PAM sequence through the PI domain. Structural studies revealed that two conserved residues (Arg^1333^ and Arg^1335^) in the PI domain form hydrogen bonds with the second and third nucleobases in the 5′-NGG-3′ PAM, respectively. A couple of SpCas9 variants have been engineered to overcome the stringent requirement for the PAM sequence. Three SpCas9 variants including D1135V/R1335Q/T1337R (VQR), D1135E/R1335Q/T1337R (EQR), and D1135V/G1218R/R1335E/T1337R (VRER) mutations were first identified via a bacterial selection system, which recognize the 5′-NGA-3′, 5′-NGAG-3′, and 5′-NGCG-3′ PAMs, respectively.^23^ Structural studies revealed that these SpCas9 variants recognized the non-canonical PAMs via an induced fit mechanism, in which the amino acid substitutions could cause conformational rearrangement of the PAM DNA.^24,25^ Subsequently, a strategy that introduces non-base-specific interactions to compensate base-specific interaction led to the identification of SpCas9-NG, a SpCas9 variant with relaxed specificity toward 5′-NG-3′ PAMs.^26^

Most recently, a SpCas9 variant (xCas9 3.7) with the broadest PAM compatibility was identified via a phage-assisted continuous evolution.^22^ Here we provide structural and biochemical evidence that R1335 stabilized by salt bridges with E1219 is important for the stringent specificity for PAM recognition. E1219V mutation in xCas9 3.7 promotes the rotamer flexibility of R1335, thus allowing xCas9 3.7 to have a broader PAM compatibility than WT SpCas9. This supplied a new strategy for structure-based engineering of CRISPR-Cas genome-editing tools to expand the targeting range. Furthermore, our data also show that either impairing the interaction between REC/ PI domain and DNA or making REC domains conformationally more flexible promotes the DNA targeting specificity of SpCas9. SpCas9 mutants engineered based on these two rationales are more specific for substrates. This mechanism appears counterintuitive, because an increased interaction between an enzyme and its substrates generally is expected to favor the selectivity of the enzyme. However, it is of note that high substrate specificity of SpCas9 should be governed by pairing with the SpCas9-bound sgRNA. In contrast with the sequence-specific interactions formed between the PI domain and PAM sequence, REC2 and REC3 domains exclusively contact with the backbone of DNA substrate. The non-sequence-specific interaction may benefit the CRISPR-Cas9 immune system for targeting different phage DNA substrates.

In summary, the data presented here reveal the structural mechanisms of broadened PAM compatibility and enhanced fidelity of xCas9 3.7. To our knowledge, this is the first report of a high-fidelity SpCas9 variant structure. Based on the structural information, new SpCas9 variants with efficiency, fidelity and PAM compatibility similar to xCas9 3.7 have been identified. Further optimization of SpCas9 can be achieved by combinations of existing variants to improve cleavage efficiency. The structural mechanisms can also be applied to Cas9 orthologs for rational engineering.

## Materials and methods

### Protein expression and purification

Point mutations were introduced using Fast Site-Directed Mutagenesis Kit in the *Streptococcus pyogenes Cas9* gene and verified by DNA sequencing. The cDNA of full-length *xCas9* was sub-cloned into the bacterial expression vector pGEX-6P-1 (GE Healthcare, with an N-terminal GST tag). WT and mutants of SpCas9 proteins were expressed in *E.coli* C43 (DE3) cells. Expression of the recombinant proteins was induced by 0.3 mM isopropyl β-D-1-thiogalactopyranoside (IPTG) at 16 °C. After overnight induction, the cells were collected by centrifugation, xCas9 was resuspended in buffer A (25 mM Tris-HCl, pH 8.0, 1 M NaCl, 3 mM DTT) supplemented with 1 mM protease-inhibitor PMSF (phenylmethanesulphonylfluoride, Sigma). The cells were subjected to lysis by sonication and cell debris was removed by centrifugation at 23,708 × g for 40 min at 4 °C. The lysate was first purified using glutathione sepharose 4B (GS4B) beads (GE Healthcare). The beads were washed and the bound proteins were cleaved by precision protease in buffer B (25 mM Tris-HCl, pH 8.0, 300 mM NaCl, 3 mM DTT) overnight at 4 °C to remove the GST tag. The cleaved xCas9 protein was eluted from GS4B resin and further fractionated by heparin sepharose column and ion exchange chromatography via FPLC (AKTA Pure, GE Healthcare).

### In vitro transcription and purification of sgRNA

The sgRNA was transcribed in vitro using T7 polymerase and purified using corresponding concentration denaturing polyacrylamide gel electrophoresis. Transcription template (dsDNA) for sgRNA was generated by PCR. Buffer containing 0.1 M HEPES-K pH 7.9, 12 mM MgCl_2_, 30 mM DTT, 2 mM Spermidine, 2 mM each NTP, 80 μg/mL home-made T7 polymerase and 500 nM transcription template was conducted for the transcription reactions. The reactions were carried out at 37 °C for 2-6 h and stopped by freezing for 1 h at −80 °C. Pyrophosphate was precipitated with Mg^2+^ at cold temperatures, and DNA templates were precipitated with Spermidine. After the precipitation was removed, RNAs was precipitated by ethanol precipitation. The RNA-containing pellets were then resuspended and purified by gel electrophoresis on a denaturing (8 M urea) polyacrylamide gel. RNA bands were excised from the gel and recovered with Elutrap System followed by ethanol precipitation. RNAs were resuspended in diethy pyrocarbonate H_2_O and stored at −80 °C.

### Reconstitution of the xCas9–sgRNA–dsDNA ternary complex

To assemble xCas9-sgRNA-dsDNA complex, dsDNA target strand and non-target strand were mixed in a 1:1.25 molar ratio (final concentration 80 μM) in annealing buffer (100 mM NaCl, 25 mM Tris-HCl, pH 8.0), hybridized by heating to 95 °C for 3 min, followed by slow cooling to room temperature. xCas9 protein was incubated with sgRNA and pre-hybridized dsDNA at the molar ratio of 1:2.5:3 at room temperature for 5 min and 4 °C for 1 h supplemented with 2 mM MgCl_2_. The complex was applied onto size-exclusion chromatography (HiLoad 16/600 Superdex 200, GE Healthcare) with buffer C (10 mM Tris-HCl, pH 8.0, 150 mM NaCl, 3 mM DTT) to remove excess sgRNA and dsDNA. Purity of the protein was monitored at all stages of the purification process using SDS-PAGE (polyAcrylamide gel electrophoresis) and visualized by Coomassie blue staining. sgRNA and dsDNA were monitored using 10% denaturing TBE-Urea and visualized by ethidium bromide staining. Purified complexes were concentrated to 10-15 mg/mL, flash frozen in liquid nitrogen, and stored at −80 °C.

### Crystallization, data collection, structure determination and refinement

Crystals of the xCas9-sgRNA-dsDNA (AAG PAM) and xCas9-sgRNA-dsDNA (GAT PAM) complexes were generated by mixing the protein complex with an equal amount of well solution (2 μL) by the hanging-drop vapour-diffusion method. Crystals of xCas9-sgRNA-dsDNA (AAG PAM) grew to their maximum size within ten days in the solution containing 0.1 M Hepes pH 7.5, 0.2 M Ammonium sulfate, 10% ispropanol, and 16% (w/v) Polyethylene glycol (PEG) 4000 at 20 °C. xCas9-sgRNA-dsDNA(GAT PAM) in the solution containing 0.1 M Hepes pH 7.5, 0.2 M Ammonium sulfate, 10% ispropanol, 16% (w/v) Polyethylene glycol (PEG) 4000, and 30% v/v ( + /-)-2-Methyl-2,4-pentanediol at 20 °C.

Before data collection, the crystals were transferred into cryo-protectant buffer (the crystallization buffer containing 20% (w/v) glycerol) and flash-cooled in liquid nitrogen. X-ray diffraction data were collected at beamline BL-19U1 at Shanghai Synchrotron Radiation Facility (SSRF) using a DECTRIS PILATUS3 6 M detector. All images were collected at the wavelength of 0.9789 Å with 1° rotation. The diffraction data for xCas9-sgRNA-dsDNA (GAT PAM) and xCas9-sgRNA-dsDNA (GAT PAM) were processed with HKL2000^27^ and XDS,^28^ respectively. The structures were determined by molecular replacement (MR) with the program PHASER.^29^ Each domain of the SpCas9 (PDB: 4OO8) was used as an individual search model for MR. The initial models were improved by several rounds of manual building in COOT^30^ and structural refinement in PHENIX.^31^ The final models were validated through MOLPROBITY^32^ and PROCHECK.^33^ Data collection and structural refinement statistics were listed in Supplementary information, Table S1. All of the structural figures were prepared using PYMOL.^34^

### In vitro cleavage assay

#### Endonuclease cleavage activity assays

Target DNA sequences were cloned into pUC18 vector using *Bam*H1 and *Eco*R1 and DNA substrates were generated from PCR assay using pUC18 primers. Target DNA sequences contain 20-nt target sequence plus 5′-TGG-3′, 5′-AGA-3′, 5′-GAT-3′ or 5′-TGT-3′ PAM motif.

#### In vitro PAM compatibility cleavage assay

Reactions were performed in a 20 μL system containing 0.5 μg Cas9, 0.1 μg sgRNA and 0.2 μg dsDNA. Cas9 and sgRNA were pre-incubated in cleavage buffer (20 mM Hepes-Na, pH 7.5, 2 mM MgCl_2_, 100 mM KCl, 1 mM dithiothreitol, 5% glycerol, 0.5 mM MnCl_2_) at room temperature for 5 min. Cleavage reactions were conducted at 37 °C for 60 min in cleavage buffer. Reactions were stopped by adding 2 × TBE-urea gel loading buffer. Cleavage products were run on TBE-urea 6% PAGE at room temperature in 1 × TBE running buffer and visualized by EB staining and quantified using ImageQuant software (GE Healthcare).

#### Fidelity cleavage assay

Fully matched and mismatched substrate DNA cleavage reactions were performed in a 20 μL system containing 0.5 μg SpCas9 variant, 0.1 μg sgRNA and 0.2 μg dsDNA. An SpCas9 variant and sgRNA were pre-incubated at room temperature for 5 min. Target DNA sequence containing 5′-TGG-3′ PAM motif cleavage reactions were conducted at 37 °C for 30 min in cleavage buffer (20 mM Hepes-Na, pH 7.5, 2 mM MgCl_2_, 100 mM KCl, 1 mM dithiothreitol, 5% glycerol, 0.5 mM MnCl_2_), and 5′-TGT-3′ PAM motif cleavage reactions were conducted at 37 °C for 60 min in cleavage buffer plus 1.5 mM MnCl_2_. Cleavage products treated as described above.

#### Time course cleavage assay

Reactions were performed in a 100 μL system containing 2.5 μg SpCas9 variant, 0.5 μg sgRNA and 1 μg dsDNA at 37 °C. An SpCas9 variant and sgRNA were pre-incubated in cleavage buffer (20 mM Hepes-Na, pH 7.5, 2 mM MgCl_2_, 100 mM KCl, 1 mM dithiothreitol, 5% glycerol, 0.5 mM MnCl_2_) at room temperature for 5 min. Cleavage reactions for substrates containing 5′-TGG-3′ or 5′-AAG-3′ PAM were stopped at indicated time points (1, 5, 15, 30, 60 min for 5′-TGG-3′ and 5, 15, 30, 60, 120 min for 5′-AAG-3′) respectively, and the samples were examined as above indicated.

### T7 endonuclease I assays for genome modification

Plasmids encoding SpCas9 variants and guide RNAs were prepared based on the pX330-U6-Chimeric_BB-CBh-hSpCas9 plasmid (Addgene plasmid #42230).^35^ 293 T cells were incubated at 37 °C for 72 h post-transfection before genomic DNA extraction. Genomic DNA was extracted using the Universal Genomic DNA Kit (CWBiotech) following the manufacturer’s protocol. The genomic region flanking the CRISPR target site was amplified by PCR (target sites and primers listed in Supplementary information, Fig. 2), and products were purified using GeneJET Gel Extraction Kit (Thermo Fisher Scientific) following the manufacturer’s protocol. A total of 250 ng purified PCR products were mixed with 2 µL 10 × NEB 2 buffer (NEB) and ultrapure water to a final volume of 20 µL, and subjected to a re-annealing process to enable heteroduplex formation: 95 °C for 3 min, 95 °C to 85 °C ramping at −2 °C/s, 85 °C to 25 °C ramping at −0.1 °C/s, and 25 °C hold for 10 min. After re-annealing, products were treated with 1 µL T7 endonuclease at 37 °C for 15 min following the manufacturer’s recommended protocol, and analyzed on TBE-urea 7% PAGE. Gels were stained with EB for 3 min and imaged with Omega Lum C. Quantification was based on relative intensities of individual bands. Indel percentage was determined by the formula, % Indel = 100 × (1- (1- fraction cleaved) ^1/2^).

### Data availability

The atomic coordinates for the atomic model of xCas9 3.7/GAT and xCas9 3.7/AAG complex have been deposited in the Protein Data Bank under accession numbers 6AEG and 6AEB. All other data are available from the corresponding authors on reasonable request.

## Supplementary information

Supplementary information, Figure S1

Supplementary information, Figure S2

Supplementary information, Figure S3

Supplementary information, Figure S4

Supplementary information, Figure S5

Supplementary information, Figure S6

Supplementary information, Table S1

Supplementary information, Table S2
