# Supplementary material for: Structural insights into a high fidelity variant of SpCas9
Source: Cell Res. 2019 Jan 21;29(3):183–92. doi: 10.1038/s41422-018-0131-6 (PMC6460432; doi:10.1038/s41422-018-0131-6)
Supplement: Supplementary file 1 — Supplementary information, Figure S1 [file 41422_2018_131_MOESM1_ESM.pdf]

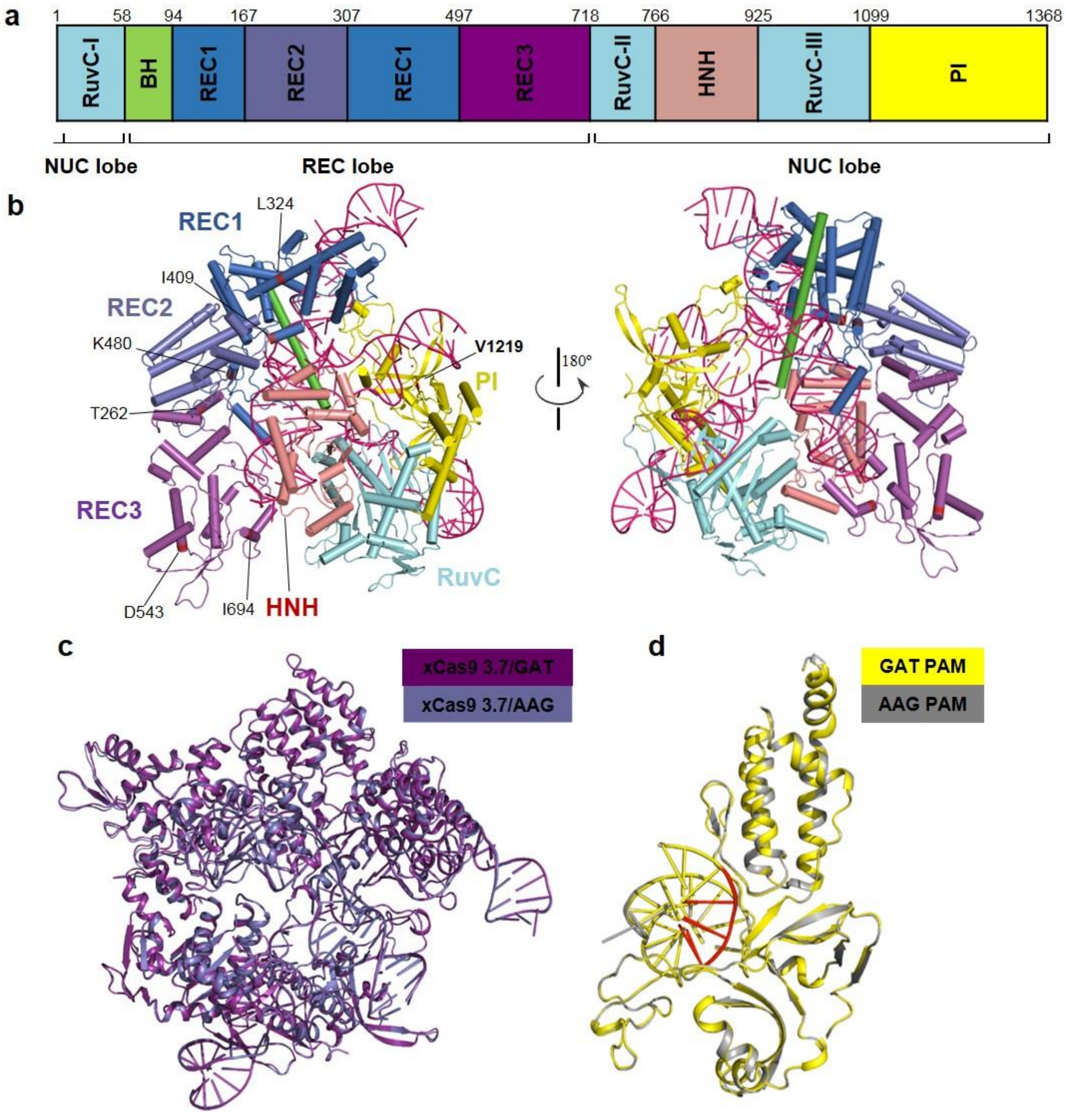

Fig. S1 Structural comparison of xCas9 3.7/GAT and xCas9 3.7/AAG

a. Domain organization of xCas9 3.7. BH, bridge helix; PI, PAM-interacting domain. Color coding is as in Fig. 1a.

b. Cartoon representation of the xCas9 3.7/sgRNA/DNA complex.

c. Superimposition of the overall structures of xCas9 3.7/GAT (purple) and xCas9 3.7/AAG (slate).

d. Structural comparison of PI domains of xCas9 3.7/GAT (yellow) and xCas9 3.7/AAG (gray)
