# Supplementary material for: Structural insights into a high fidelity variant of SpCas9
Source: Cell Res. 2019 Jan 21;29(3):183–92. doi: 10.1038/s41422-018-0131-6 (PMC6460432; doi:10.1038/s41422-018-0131-6)
Supplement: Supplementary file 2 — Supplementary information, Figure S2 [file 41422_2018_131_MOESM2_ESM.pdf]

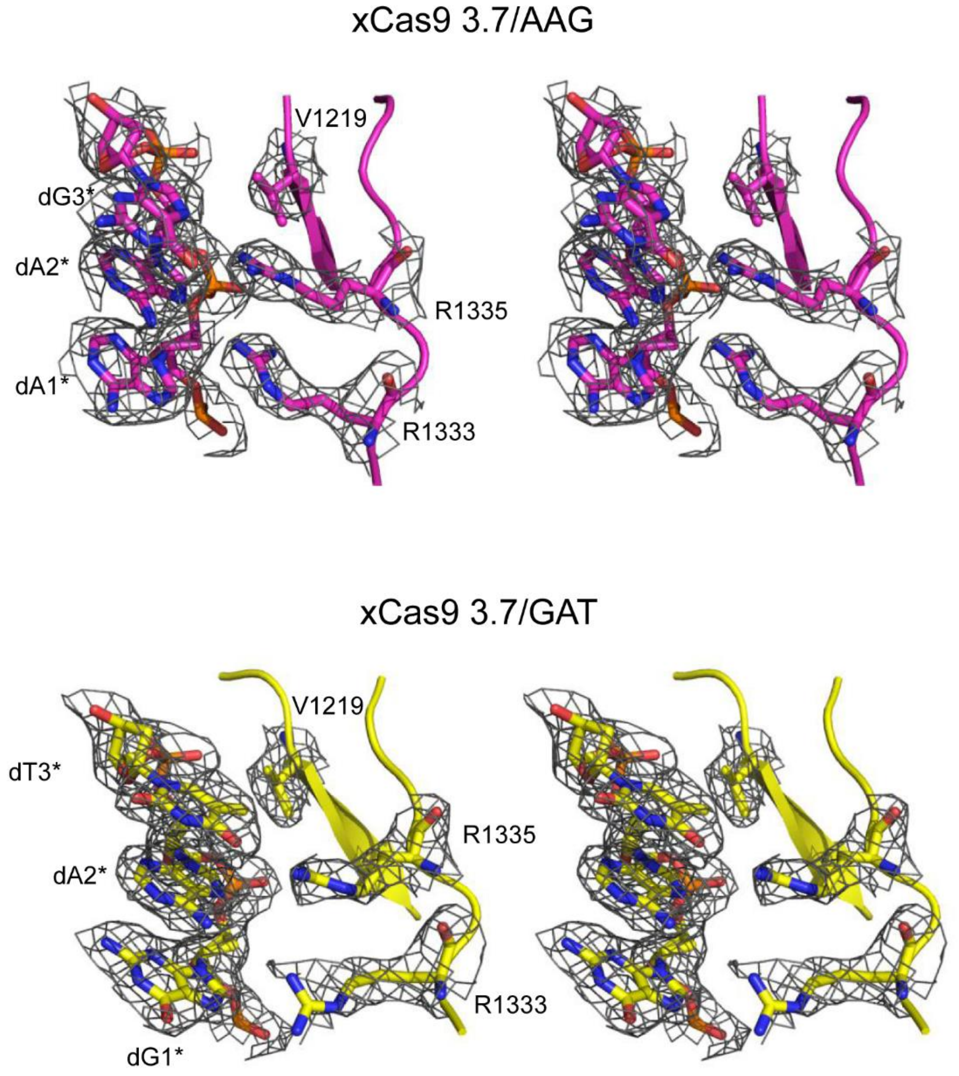

Fig. S2 Electron density maps

The 2*Fo* – *Fc* omit electron density maps for R1333, R1335, V1219 and the PAM nucleotides in xCas9 3.7/GAT and xCas9 3.7/AAG are shown as a gray mesh (contoured at 1.0 σ).
