# Supplementary figures and images for: Structural insights into a high fidelity variant of SpCas9
Source: Cell Res. 2019 Jan 21;29(3):183–92. doi: 10.1038/s41422-018-0131-6 (PMC6460432; doi:10.1038/s41422-018-0131-6)

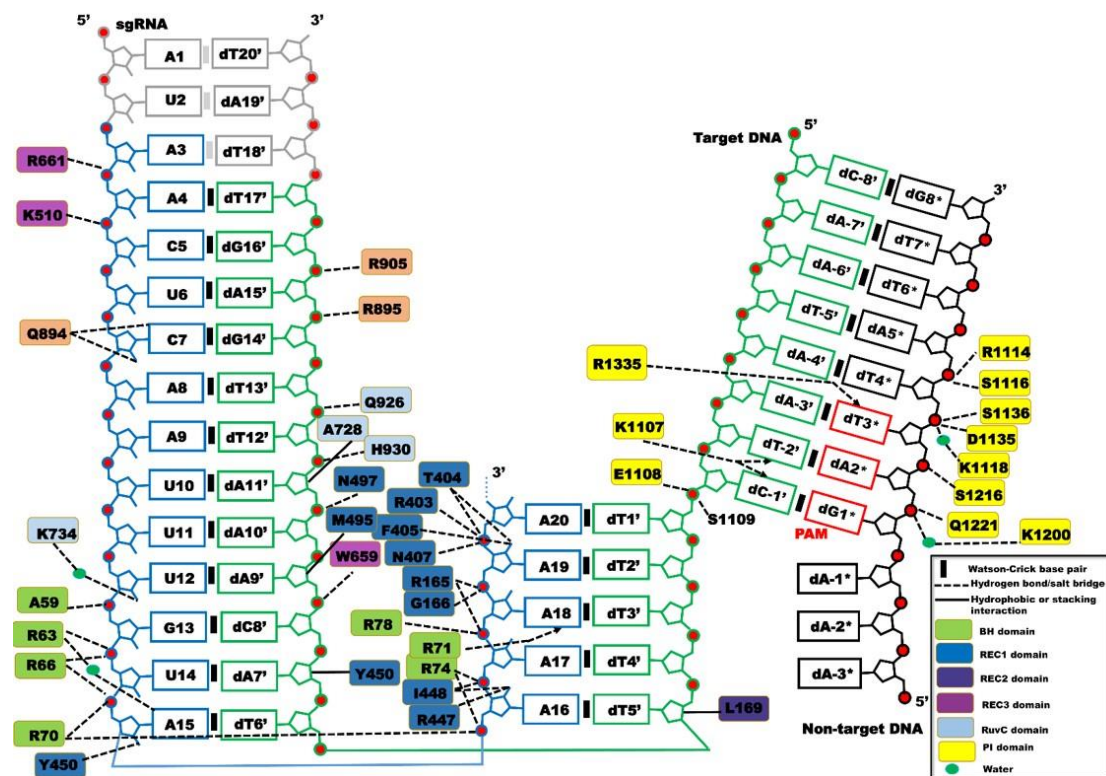

Fig. S3 Schematic representation of interactions of sgRNA/DNA heteroduplex with xCas9 3.7

Supplement: Supplementary file 3 — Supplementary information, Figure S3 [file 41422_2018_131_MOESM3_ESM.pdf]

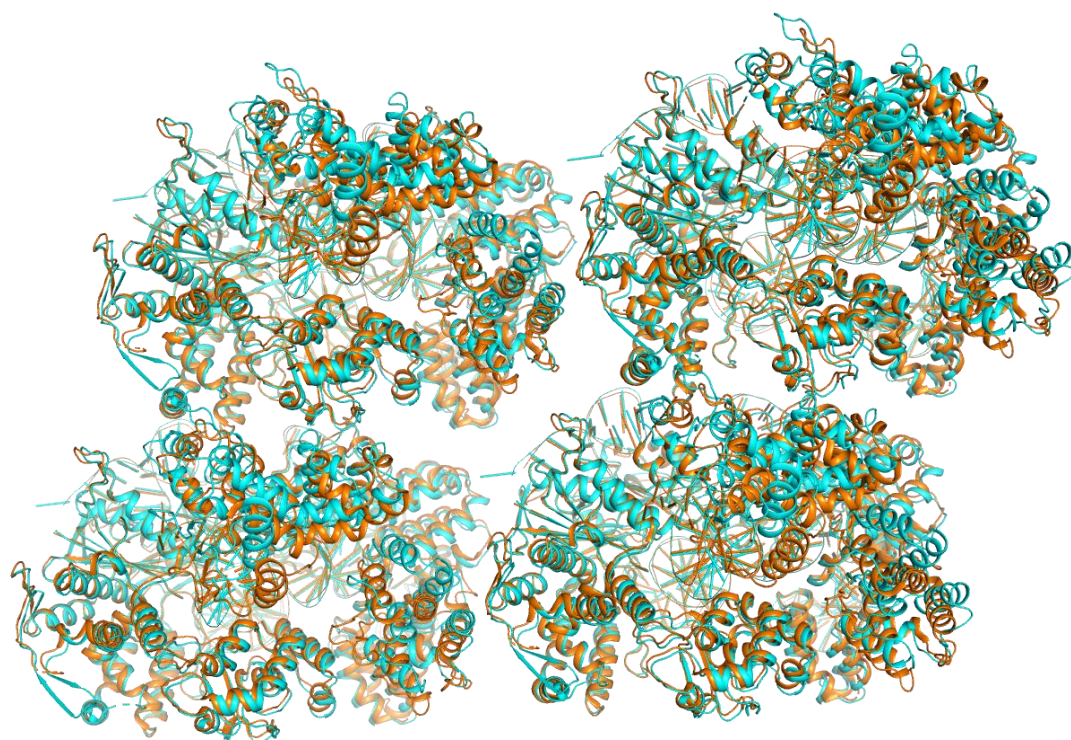

**Fig. S4 Crystal packing of SpCas9-sgRNA-DNA (PDB: 4UN3) (Orange) and xCas9 3.7 (Cyan)**

Supplement: Supplementary file 4 — Supplementary information, Figure S4 [file 41422_2018_131_MOESM4_ESM.pdf]
