# Supplementary material for: Structural insights into a high fidelity variant of SpCas9
Source: Cell Res. 2019 Jan 21;29(3):183–92. doi: 10.1038/s41422-018-0131-6 (PMC6460432; doi:10.1038/s41422-018-0131-6)
Supplement: Supplementary file 5 — Supplementary information, Figure S5 [file 41422_2018_131_MOESM5_ESM.pdf]

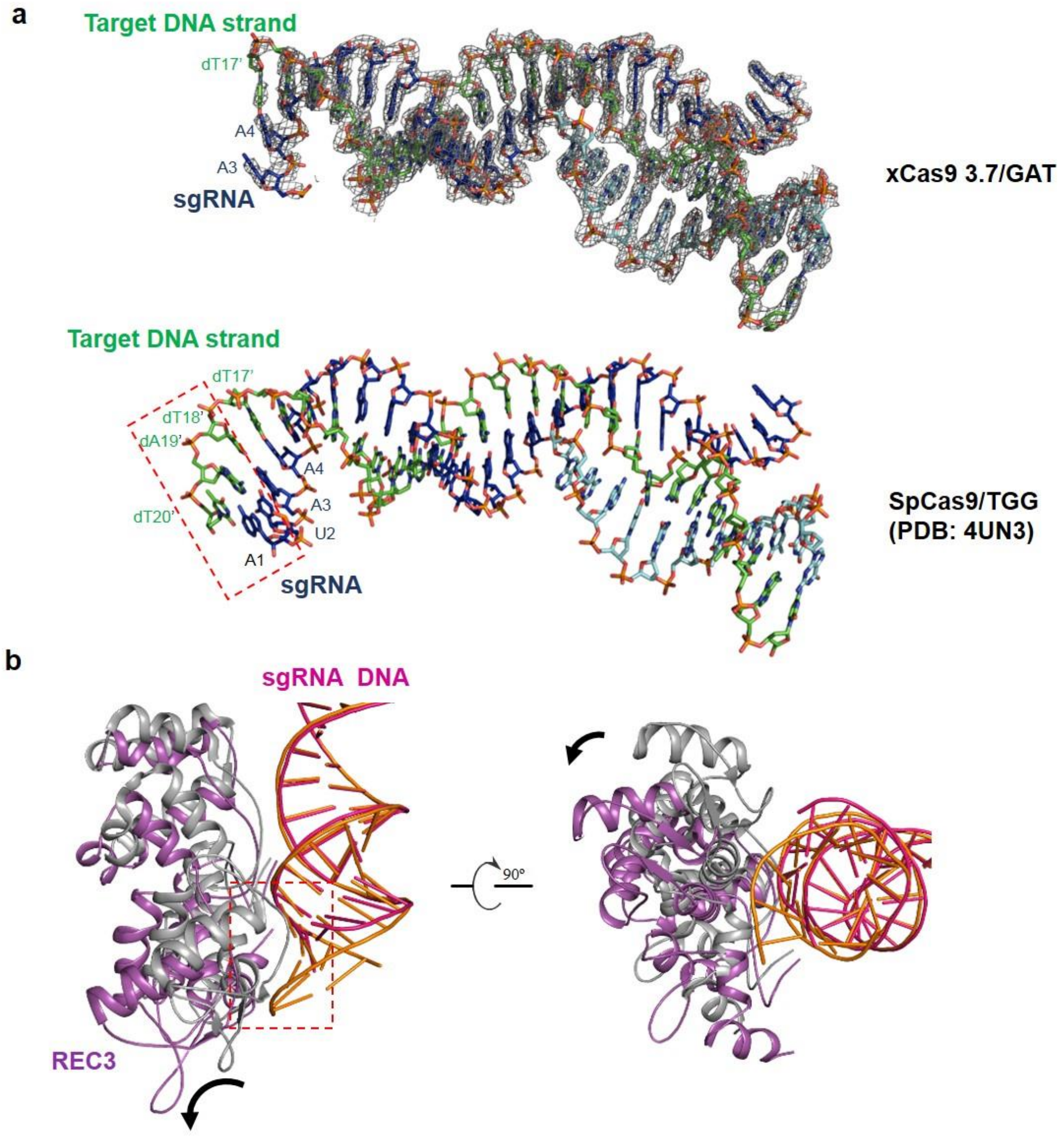

Fig. S5 Structures of sgRNA/DNA heteroduplex bound by xCas9 3.7 and SpCas9

a. Structural comparison of xCas9 3.7- and SpCas9-bound sgRNA/DNA heteroduplex. Top panel: Shown in mesh (gray) is the electron density "$2Fo - Fc$" of xCas9 3.7-bound sgRNA/DNA heteroduplex contoured at 1.2 σ. Bottom panel: structure of sgRNA/DNA heteroduplex bound by WT SpCas9 (PDB: 4UN3).

b. Superimposition of the interfaces of REC3-RNA/DNA heteroduplex of xCas9 3.7 (purple) and SpCas9 (gray) in two orientations. The xCas9 3.7- and SpCas9 (gray)-bound sgRNA/DNA are colored hot pink and orange, respectively.
