# Supplementary material for: Structural insights into a high fidelity variant of SpCas9
Source: Cell Res. 2019 Jan 21;29(3):183–92. doi: 10.1038/s41422-018-0131-6 (PMC6460432; doi:10.1038/s41422-018-0131-6)
Supplement: Supplementary file 6 — Supplementary information, Figure S6 [file 41422_2018_131_MOESM6_ESM.pdf]

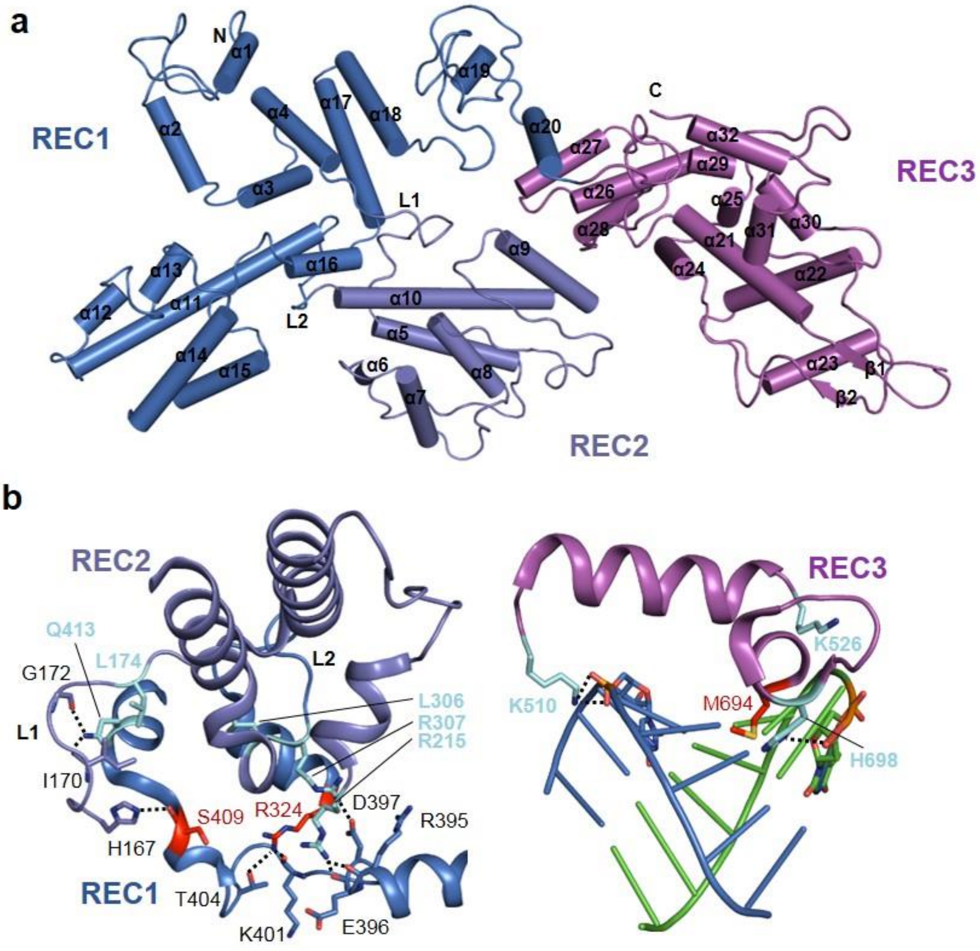

Fig. S6 The interfaces of REC1-REC2 (HFH1) and REC3-RNA/DNA heteroduplex (HFH2) of SpCas9 (PDB: 4UN3)

a. The overall structure of REC1 (blue), REC2 (slate) and REC3 (purple) domains. The secondary structural elements in the structure are numbered.

b. The interfaces of REC1-REC2 and REC3-RNA/DNA heteroduplex of SpCas9 (PDB: 4UN3) with the location of rationally designed mutations shown.
