# Supplementary material for: Structural insights into a high fidelity variant of SpCas9
Source: Cell Res. 2019 Jan 21;29(3):183–92. doi: 10.1038/s41422-018-0131-6 (PMC6460432; doi:10.1038/s41422-018-0131-6)
Supplement: Supplementary file 7 — Supplementary information, Table S1 [file 41422_2018_131_MOESM7_ESM.pdf]

Supplementary information, Table S1 | **Data collection and refinement statistics**

	xCas9 3.7/GAT	xCas9 3.7/AAG
Data collection		
Beam Line	BL19U, SSRF	BL19U, SSRF
Space Group	*C*2	*C*2
Unit Cell Parameters		
a, b, c (Å)	190.48, 70.46, 183.53	361.85, 71.13, 198.32
α, β, γ (˚)	90.00, 106.48, 90.00	90.00, 101.75, 90.00
Wavelength(Å)	0.9789	0.9789
Resolution limits(Å)	50.00-2.70(2.75-2.70)	50.00-3.00(3.18-3.00)
No. of unique reflections	60687(3085)	98977(15638)
Completeness (%)	94.1(96.1)	99.3(98.0)
Redundancy	2.8(2.6)	4.2(3.9)
R_{merge} (%) [a]	8.2(45.0)	11.9(97.3)
Mean I/σ(I)	9.1(1.8)	10.1(1.6)
Refinement		
Resolution limits(Å)	45.00-2.70	50.00-3.00
No. reflections	56285	98522
R_{work}(%) [b]/R_{free}(%) [c]	21.47/26.25	23.10/28.28
R.m.s.d for bonds (Å)	0.005	0.004
R.m.s.d for angles (˚)	0.762	0.698
Averaged B factor of the structure	37.1	73.3
No. of non-hydrogen protein atoms	13426	26372
Ramachandran plot (%)		
Preferred region	93.45	90.28
Allowed region	6.55	9.72
Outliers	0.00	0.00

Highest-resolution shell is shown in parenthesis.

[a] $R_{merge} = \sum|I_i - \langle I\rangle|/\sum|I|$, where I_i is the intensity of an individual reflection and $\langle I\rangle$ is the average intensity of that reflection.

[b] $R_{work} = \sum||F_o| - |F_c||/\sum|F_o|$, where F_o and F_c are the observed and calculated structure factors for reflections, respectively.

[c] R_{free} was calculated as R_{work} using the 5% of reflections that were selected randomly and omitted from refinement.
