# Supplementary material for: Structural insights into a high fidelity variant of SpCas9
Source: Cell Res. 2019 Jan 21;29(3):183–92. doi: 10.1038/s41422-018-0131-6 (PMC6460432; doi:10.1038/s41422-018-0131-6)
Supplement: Supplementary file 8 — Supplementary information, Table S2 [file 41422_2018_131_MOESM8_ESM.pdf]

Supplementary Table 2 | **Nucleic acid sequences used in the study**

**RNA sequence**	**Sequence (5' to 3')**
SpCas9_sgRNA	GGAUAACUCAAUUUGUAAAAAAGUUUUAGAGCUAGAAAUAGCAAGUUAAAAUAAGGCUAGUCCGUUAUCAACUUGAAAAAGUGGCACCGAGUCGGUGCUU
**dsDNA substrate sequence**	
TarDNA_AAG-PAM	CAATACTTTTTTTTACAAATTGAGTTAT
Non-tarDNA_AAG-PAM	AAAAAGTATTG
TarDNA_GAT-PAM	CAATAATCTTTTTTACAAATTGAGTTAT
Non-tarDNA_GAT-PAM	AAAGATTATTG
**RNA transcription assay DNA template**	
SpCas9_sgRNA_DNA template	ATGTAATACGACTCACTATAGGATAACTCAATTTGTAAAAAAGTTTTAGAGCTAGAAATAGCAAGTTAAAATAAGGCTAGTCCGTTATCAACTTGAAAAAGTGGCACCGAGTCGGTGCTT
***In vitro* DNA cleavage assay primer**	
TGG_BamH1_5	GATCCCAATTCCATTTTTTTACAAATTGAGTTATG
TGG_EcoR1_3	AATTCATAACTCAATTTGTAAAAAATGGAATTGG
AGA_BamH1_5	GATCCCAATTTCTTTTTTTACAAATTGAGTTATG
AGA_EcoR1_3	AATTCATAACTCAATTTGTAAAAAAAGAAATTGG
GAT_BamH1_5	GATCCCAATTATCTTTTTTACAAATTGAGTTATG
GAT_EcoR1_3	AATTCATAACTCAATTTGTAAAAAAGATAATTGG
TGT_BamH1_5	GATCCCAATTACATTTTTTTACAAATTGAGTTATG
TGT_EcoR1_3	AATTCATAACTCAATTTGTAAAAAATGTAATTGG
TGG_13/14_BamH1_5	GATCCCAATTCCATTTTTTTACAAATCAAGTTATG
TGG_13/14_EcoR1_3	AATTCATAACTTGATTTGTAAAAAATGGAATTGG
TGG_15/16_BamH1_5	GATCCCAATTCCATTTTTTTACAAATTGGATTATG
TGG_15/16_EcoR1_3	AATTCATAATCCAATTTGTAAAAAATGGAATTGG

TGG_17/18_BamH1_5	GATCCCAATTCCATTTTTTACAAATTGAGCCATG
TGG_17/18_EcoR1_3	AATTCATGGCTCAATTTGTAAAAAATGGAATTGG
TGG_18/19_BamH1_5	GATCCCAATTCCATTTTTTACAAATTGAGTCGTG
TGG_18/19_EcoR1_3	AATTCACGACTCAATTTGTAAAAAATGGAATTGG
TGT_13/14_BamH1_5	GATCCCAATTACATTTTTTACAAATCAAGTTATG
TGT_13/14_EcoR1_3	AATTCATAACTTGATTTGTAAAAAATGTAATTGG
TGT_15/16_BamH1_5	GATCCCAATTACATTTTTTACAAATTGGATTATG
TGT_15/16_EcoR1_3	AATTCATAATCCAATTTGTAAAAAATGTAATTGG
TGT_17/18_BamH1_5	GATCCCAATTACATTTTTTACAAATTGAGCCATG
TGT_17/18_EcoR1_3	AATTCATGGCTCAATTTGTAAAAAATGTAATTGG
TGT_18/19_BamH1_5	GATCCCAATTACATTTTTTACAAATTGAGTCGTG
TGT_18/19_EcoR1_3	AATTCACGACTCAATTTGTAAAAAATGTAATTGG
pUC18_5	CATTCAGGCTGCGCAACTGTTGG
pUC18_3	CCAGGCTTTACACTTTATGCT
***In vitro* cleavage assay DNA sequence**	
pUC18_substrate	CATTCAGGCTGCGCAACTGTTGGGAAGGGCGATCGGTGCGGGCCTCTTCGCTATTACGCCAGCTGGCGAAAGGGGGATGTGCTGCAAGGCGATTAAGTTGGGTAACGCCAGGGTTTTCCCAGTCACGACGTTGTAAAACGACGGCCAGTGCCAAGCTTGCATGCCTGCAGGTCGACTCTAGAGGATCCCAATTCCATTTTTTACAAATTGAGTTATGAATTCGTAATCATGGTCATAGCTGTTTCCTGTGTGAAATTGTTATCCGCTCACAATTCCACACAACATACGAGCCGGAAGCATAAAGTGTAAAGCCTGG
***EMX1* target site Sequence**	GTCACCTCCAATGACTAGGGTGG
***EMX1* target site mismatched primer**	
TGG_15/16_5	caccGTCAGGTCCAATGACTAGGG

TGG_15/16_3	aaacCCCTAGTCATTGGACCTGAC
TGG_17/18_5	caccGTGTCCTCCAATGACTAGGG
TGG_17/18_3	aaacCCCTAGTCATTGGAGGACAC
TGG_18/19_5	caccGAGACCTCCAATGACTAGGG
TGG_18/19_3	aaacCCCTAGTCATTGGAGGTCTC
TGG_wt_5	caccGTCACCTCCAATGACTAGGG
TGG_wt_3	aaacCCCTAGTCATTGGAGGTGAC
***VEGFA* target site Sequence**	GGGAAGCTGGGTGAATGGAGCGA
***VEGFA* target site mismatched primer**	
CGA_15/16_5	caccGGGATCCTGGGTGAATGGAG
CGA_15/16_3	aaacCTCCATTCACCCAGGATCCC
CGA_17/18_5	caccGGCTAGCTGGGTGAATGGAG
CGA_17/18_3	aaacCTCCATTCACCCAGCTAGCC
CGA_18/19_5	caccGCCAAGCTGGGTGAATGGAG
CGA_18/19_3	aaacCTCCATTCACCCAGCTTGGC
CGA_wt_5	caccGGGAAGCTGGGTGAATGGAG
CGA_wt_3	aaacCTCCATTCACCCAGCTTCCC
***FANCF* target site Sequence**	GCGGTCTCAAGCACTACCTACGT
***FANCF* target site mismatched primer**	
CGT_15/16_5	caccGCGGAGTCAAGCACTACCTA
CGT_15/16_3	aaacTAGGTAGTGCTTGACTCCGC
CGT_17/18_5	caccGCCCTCTCAAGCACTACCTA
CGT_17/18_3	aaacTAGGTAGTGCTTGAGAGGGC
CGT_18/19_5	caccGGCGTCTCAAGCACTACCTA
CGT_18/19_3	aaacTAGGTAGTGCTTGAGACGCC
CGT_wt_5	caccGCGGTCTCAAGCACTACCTA
CGT_wt_3	aaacTAGGTAGTGCTTGAGACCGC
**T7 endonuclease I assay primer**	
EMX1 test F	CCATCCCCTTCTGTGAATGT
EMX1 test R	GGAGATTGGAGACACGGAGA
VEGFA test F	CCCCTTTCCAAAGCCCATTCC
VEGFA test R	TCTGCGGACGCTCAGTGAAG
FANCF test F	GTTGCCCAGAGTCAAGGAAC
FANCF test R	CAGGTGGTAACGAGCTGCATC

**T7 endonuclease I assay DNA sequence**	
EMX1 target site flanking sequence	GGAGATTGGAGACACGGAGAGCAGCTG<br>GGAGGCTGCGGTGGCGGGCGGGCCCGC<br>CCAGGCAGGCAGGCTCTCCGAGGAGAA<br>GGCCAAGTGGTCCCAGGCCTCAGCCAGC<br>CCATTGCTTGTCCCTCTGTCAATGGCGGC<br>CCCGGGCTTCAAGCCCTGTGGGGCCATG<br>ACTCCAGGCCTCCCCAAAGCCTGGCCAG<br>GGAGTGGCCAGAGTCCAGCTTGGGCCCA<br>CGCAGGGGCCTGGCCAGCAGCAAGCAG<br>CACTCTGCCCTCGTGGGTTTGTGGTTGCC<br>CACCCTAGTCATTGGAGGTGACATCGAT<br>GTCCTCCCCATTGGCCTGCTTCGTGGCAA<br>TGCGCCACCGGTTGATGTGATGGGAGCC<br>CTTCTTCTTCTGCTCGGACTCAGGCCCTT<br>CCTCCTCCAGCTTCTGCCGTTTGTACTTT<br>GTCCTCCGGTTCTGGAACCACACCTTCA<br>CCTGGGCCAGGGAGGGAGGGGCACAGA<br>TGAGAAACTCAGGAGGCCCCCAGAGCA<br>GCCACTGGGGCCTCAACACTCAGGCTGA<br>GCTGAGAGCCTGATGGGAAGACTGAGG<br>CTACATAGGGTTAGGGGCCCCAGGCCGG<br>GGTCCCCTCTGACCAGCTGCTCCCATGG<br>GTCTAACATTCACAGAAGGGGATGG
VEGFA target site flanking sequence	CCCCTTTCCAAAGCCCATTCCCTCTTTAG<br>CCAGAGCCGGGGTGTGCAGACGGCAGTC<br>ACTAGGGGGCGCTCGGCCACCACAGGG<br>AAGCTGGGTGAATGGAGCGAGCAGCGT<br>CTTCGAGAGTGAGGACGTGTGTGTCTGT<br>GTGGGTGAGTGAGTGTGTGCGTGTGGGG<br>TTGAGGGCGTTGGAGCGGGGAGAAGGC<br>CAGGGGTCACTCCAGGATTCCAATAGAT<br>CTGTGTGTCCCTCTCCCCACCCGTCCCTG<br>TCCGGCTCTCCGCCTTCCCCTGCCCCCTT<br>CAATATTCCTAGCAAAGAGGGAACGGCT<br>CTCAGGCCCTGTCCGCACGTAACCTCAC<br>TTTCCTGCTCCCTCCTCGCCAATGCCCCG<br>CGGGCGCGTGTCTCTGGACAGAGTTTCC<br>GGGGGCGGATGGGTAATTTTCAGGCTGT<br>GAACCTTGGTGGGGGTCGAGCTTCCCCT<br>TCATTGCGGCGGGCTGCGGGCCAGGCTT<br>CACTGAGCGTCCGCAGA
FANCF target site flanking sequence	GTTGCCCAGAGTCAAGGAACACGGATAA<br>AGACGCTGGGAGATTGACATGCATTTCG

	ACCAATAGCATTGCAGAGAGGCGTATCA<br>TTTCGCGGATGTTCCAATCAGTACGCAG<br>AGAGTCGCCGTCTCCAAGGTGAAAGCGG<br>AAGTAGGGCCTTCGCGCACCTCATGGAA<br>TCCCTTCTGCAGCACCTGGATCGCTTTTC<br>CGAGCTTCTGGCGGTCTCAAGCACTACC<br>TACGTCAGCACCTGGGACCCCGCCACCG<br>TGCGCCGGGCCTTGCAGTGGGCGCGCTA<br>CCTGCGCCACATCCATCGGCGCTTTGGT<br>CGGCATGGCCCCATTCGCACGGCTCTGG<br>AGCGGCGGCTGCACAACCAGTGGAGGC<br>AAGAGGGCGGCTTTGGGCGGGGTCCAGT<br>TCCGGGATTAGCGAACTTCCAGGCCCTC<br>GGTCACTGTGACGTCCTGCTCTCTCTGCG<br>CCTGCTGGAGAACCGGGCCCTCGGGGAT<br>GCAGCTCGTTACCACCTG
